# Association of Increased Grain Iron and Zinc Concentrations with Agro-morphological Traits of Biofortified Rice

**DOI:** 10.3389/fpls.2016.01463

**Published:** 2016-09-28

**Authors:** Laura T. Moreno-Moyano, Julien P. Bonneau, José T. Sánchez-Palacios, Joseph Tohme, Alexander A. T. Johnson

**Affiliations:** ^1^School of BioSciences, The University of Melbourne, MelbourneVIC, Australia; ^2^International Center for Tropical AgricultureCali, Colombia

**Keywords:** biofortification, nicotianamine synthase, *OsNAS2*, overaccumulation, panicle, magnesium, yield

## Abstract

Biofortification of rice (*Oryza sativa* L.) with micronutrients is widely recognized as a sustainable strategy to alleviate human iron (Fe) and zinc (Zn) deficiencies in developing countries where rice is the staple food. Constitutive overexpression of the rice nicotianamine synthase (*OsNAS*) genes has been successfully implemented to increase Fe and Zn concentrations in unpolished and polished rice grain. Intensive research is now needed to couple this high-micronutrient trait with high grain yields. We investigated associations of increased grain Fe and Zn concentrations with agro-morphological traits of backcross twice second filial (BC_2_F_2_) transgenic progeny carrying *OsNAS1* or *OsNAS2* overexpression constructs under *indica/japonica* and *japonica/japonica* genetic backgrounds. Thirteen agro-morphological traits were evaluated in BC_2_F_2_ transgenic progeny grown under hydroponic conditions. Concentrations of eight mineral nutrients (Fe, Zn, copper, manganese, calcium, magnesium, potassium, and phosphorus) in roots, stems/sheaths, non-flag leaves, flag leaves, panicles, and grain were also determined. A distance-based linear model (DistLM) was utilized to extract plant tissue nutrient predictors accounting for the largest variation in agro-morphological traits differing between transgenic and non-transgenic progeny. Overall, the BC_2_F_2_ transgenic progeny contained up to 148% higher Fe and 336% higher Zn concentrations in unpolished grain compared to non-transgenic progeny. However, unpolished grain concentrations surpassing 23 μg Fe g^-1^ and 40 μg Zn g^-1^ in BC_2_F_2_ *indica/japonica* progeny, and 36 μg Fe g^-1^ and 56 μg Zn g^1^ in BC_2_F_2_ *japonica/japonica* progeny, were associated with significant reductions in grain yield. DistLM analyses identified grain-Zn and panicle-magnesium as the primary nutrient predictors associated with grain yield reductions in the *indica/japonica* and *japonica/japonica* background, respectively. We subsequently produced polished grain from high-yield BC_2_F_2_ transgenic progeny carrying either the *OsNAS1* or *OsNAS2* overexpression constructs. The *OsNAS2* overexpressing progeny had higher percentages of Fe and Zn in polished rice grain compared to the *OsNAS1* overexpressing progeny. Results from this study demonstrate that genetic background has a major effect on the development of Fe and Zn biofortified rice. Moreover, our study shows that high-yielding rice lines with Fe and Zn biofortified polished grain can be developed by *OsNAS2* overexpression and monitoring for Zn overaccumulation in the grain.

## Introduction

Over two billion people worldwide suffer from micronutrient deficiencies due to a lack of essential vitamins and minerals in their diet^1^. Iron (Fe) and zinc (Zn) deficiencies are the most widespread human micronutrient deficiencies and are particularly prevalent in resource-poor countries where there is a heavy dietary reliance on staple crops (Sands et al., 2009). Rice (*Oryza sativa* L.) is the principal source of calorie intake for about half of the world’s population. The potential of biofortified rice for alleviating widespread micronutrient malnutrition in the world’s major rice consuming countries is now widely recognized. Genetic engineering is considered a valuable strategy to alleviate Fe deficiency in rice based societies, as increases of Fe concentrations in transgenic polished rice grain have exceeded levels achieved by conventional breeding (Johnson et al., 2011; Lee et al., 2012; Trijatmiko et al., 2016). Nonetheless, the development of Fe and Zn biofortified rice is challenging. This is due to the complexity of genetic and metabolic networks controlling the homeostasis of Fe and Zn (Stomph et al., 2009; Bashir et al., 2010; Ishimaru et al., 2011; Sinclair and Krämer, 2012; Sperotto et al., 2012a). Moreover, differences in the use efficiency of Fe and Zn, variability of their concentration level in the grain and genotype-dependent source-sink relations can affect internal mobilization patterns of these micronutrients (Sperotto, 2013). Such factors constitute a challenge for the selection of suitable genetic backgrounds to maximize Fe and Zn accumulation in the grain.

In addition to being essential mineral nutrients for humans, Fe and Zn are essential elements for plants and their homeostasis processes such as coordination of uptake, buffering, translocation, and storage are tightly regulated within narrow physiological limits which promote proper plant growth and development (Grotz and Guerinot, 2006; Teklić et al., 2013). Iron is involved in electron-transfer reactions and has a key role in photosynthesis, respiration and chlorophyll biosynthesis (Hell and Stephan, 2003; Kim and Guerinot, 2007). Zinc is an important cofactor in gene transcription and the coordination of protein, nucleic acid, carbohydrate and lipid metabolism (Grotz and Guerinot, 2006; Ishimaru et al., 2011). Moreover, Fe and Zn nutrition in graminaceous and non-graminaceous plants is influenced by essential macronutrients including phosphorus, sulfur and nitrogen through cross-talks between the signaling pathways that integrate the complex metal homeostasis networks (Kutman et al., 2011; Briat et al., 2015). Deficiencies or excesses of Fe and Zn in the plant can generate major physiological disorders by internal imbalances of these micronutrients and other essential nutrients, thereby impacting plant growth and development.

Increased production of the chelating molecules nicotianamine (NA) and 2’-deoxymugineic acid (DMA) has been a popular strategy to enhance Fe and Zn uptake, internal transport, and loading into the rice grain (Suzuki et al., 2008; Lee et al., 2009, 2011; Masuda et al., 2009; Wirth et al., 2009; Zheng et al., 2010; Johnson et al., 2011; Trijatmiko et al., 2016). Nicotianamine, synthesized by nicotianamine synthase (NAS), is a structural analog and biosynthetic precursor of mugineic acid family phytosiderophores (MAs) such as DMA (Reichman and Parker, 2002). Nicotianamine facilitates the internal transport of essential trace metal cations including Fe^3+^, Fe^2+^, and Zn^2+^ (von Wiren et al., 1999; Takahashi et al., 2003). DMA is the type of MAs produced in rice as part of the Strategy-II Fe acquisition in graminaceous plants, and is involved in the solubilization of Fe^3+^ in the rhizosphere (Bashir et al., 2006). In rice, there are three NA synthase (*OsNAS)* genes expressed in cells involved in uptake and long distance transport of Fe (Inoue et al., 2003). Constitutive overexpression of the *OsNAS2* gene in transgenic rice has led to increased secretion of DMA into the rhizosphere as well as significantly increased NA and DMA concentrations in grain that are positively correlated with increased Fe and Zn concentrations in grain (Zheng et al., 2010; Johnson et al., 2011; Lee et al., 2011; Nozoye et al., 2014).

Constitutive overexpression of the *OsNAS* genes has also induced elevated concentrations of Fe and Zn in rice vegetative tissues, and in some cases this has been associated with reductions in plant grain yield. Constitutive overexpression of the *OsNAS1* gene under transcriptional control of the maize ubiquitin promoter led to 2-fold higher Fe and 6-fold higher Zn concentration in leaf tissues of 5-days old rice seedlings (Zheng et al., 2010). Activation tagging of the *OsNAS2* gene led to 2.4-fold and 1.6-fold higher Zn concentration in shoots and roots of 8-days old seedlings (Lee et al., 2011). Increased Fe concentrations in leaves were associated with reduced plant height and grain number of transgenic rice plants overexpressing the *OsNAS2* gene (Lee et al., 2012; Nozoye et al., 2014). Further studies on the effects of increased Fe and Zn concentrations by *OsNAS* overexpression on rice yield performance and internal nutrient status is required for the development of high-yielding Fe and Zn biofortified rice varieties.

As a component of Fe and Zn biofortified rice research, we have investigated the association of high grain Fe and Zn with a range of agro-morphological traits of backcross twice second filial (BC_2_F_2_) rice progeny containing *OsNAS* overexpression constructs and under two contrasting genetic backgrounds *indica/japonica* and *japonica/japonica*. To identify associations between nutrient concentrations at the whole-plant level and agro-morphological traits, we evaluated thirteen agro-morphological traits under hydroponic growth conditions and assessed concentrations of Fe, Zn, copper (Cu), manganese (Mn), calcium (Ca), magnesium (Mg), potassium (K), and phosphorus (P) in the root, stem/sheath, non-flag leaf, flag leaf, and unpolished rice grain. The concentration of Fe and Zn in polished rice grain of high-yield transgenic plants carrying either *OsNAS1* or *OsNAS2* overexpression constructs was also evaluated. The results reveal strategies to develop high-yielding Fe and Zn biofortified rice varieties carrying *OsNAS* overexpression constructs.

## Materials and Methods

### Plant Material

Backcrosses were performed at CIAT (Cali, Colombia) using single copy cv. Nipponbare rice T-DNA transformants carrying CaMV35S::*OsNAS1* (OE-*OsNAS1*) or CaMV35::*OsNAS2* (OE-*OsNAS2*) constructs (Johnson et al., 2011) as donor parents, and *indica* rice cv. IR64 and *tropical japonica* rice cv. Esperanza as recurrent parents, to yield BC_2_F_2_ progeny. BC_2_F_2_ progeny derived from crosses between OE-*OsNAS1* or OE-*OsNAS2* × IR64, are referred to as the OE-*OsNAS*/IR64 progeny hereafter. BC_2_F_2_ progeny derived from OE-*OsNAS1* or OE-*OsNAS2* × Esperanza, are referred to as the OE-*OsNAS*/Esp progeny hereafter. Null segregants from each progeny are referred to as NS progeny hereafter.

### Plant Culture and Growth Conditions

Rice grain were surfaced-sterilized with 70% ethyl alcohol and 5% sodium hypochlorite and then germinated on filter paper soaked with 7 ml of distilled water for 7 days in a controlled environment chamber at 25°C with 12-h photoperiod. After 8 days, seedlings were transplanted into a hydroponic set-up consisting of a 12 L culture box filled with Yoshida’s hydroponic solution (Yoshida, 1981) and placed in an environmentally controlled growth room. Growth conditions consisted of 12-h photoperiod with an average light intensity of 300 mmol^-1^ s^-1^ illumination (PAR) at plant level provided by 12 Phillips incandescent lamps, 60/80% day/night relative humidity and 28/24°C day/night temperature. Plants were transferred from lids of 40 holes of 25 mm in diameter to lids with 20 holes of 50 mm in diameter (Supplementary Figure 1). Nine-week-old plants were transferred from 12 L culture boxes to 20 L culture boxes to adjust the rooting volume to plant development. The Yoshida’s nutrient solution contained in full strength 40 mg N L^-1^ (as NH_4_NO_3_), 10 mg P L^-1^ (as NaH_2_ PO_4_ 2H_2_O), 40 mg K L^-1^ (as K_2_SO_4_), 40 mg Ca L^-1^ (as CaCl_2_), 40 mg Mg L^-1^ (as MgSO_4_ 7H_2_O), 0.5 mg Mn L^-1^ (as MnCl_2_ 4H_2_O), 0.05 mg Mo L^-1^ [as (NH_4_)_6_ MO_7_O_24_ 4H_2_O], 0.54 mg B L^-1^ (as H_3_BO_3_), 0.01 Zn mg L^-1^ (as ZnSO_4_ 7H_2_O), 0.01 mg Cu L^-1^ (as CuSO_4_ 5H_2_O), 2 mg Fe L^-1^ [as FeCl_3_ 6H_2_O (in mono-hydrate citric acid)] with a pH of 5.8. The strength of the nutrient solution was gradually increased in 5-day intervals from 25% (8- to 12-days old plants) over 50% (13- to 17-days old plants) to 100% (18-days old plants to maturity). From day 18, the culture solution was changed once a week and adjusted every other day to pH 5.8 with 5M NaOH.

### Transgene Detection and Experimental Design

Total genomic DNA was isolated from ∼100 mg of 3-week-old leaf tissue using the Extract-N-Amp^TM^ Tissue PCR kit (Sigma–Aldrich). To determine presence/absence of the transgene, a pair of PCR primers was designed to amplify a 1079 bp fragment of the OE-*OsNAS* constructs. The primer sequences were as follows: forward primer 5′ acaagaaagctgggtcgaat 3′ and reverse primer 5′ gcgccaagctatcaaacaag 3′. PCRs were carried out using 2 μl of DNA extract and 0.5 units of MyTaq^TM^ DNA polymerase from Bioline, 6 μl of MyTaq^TM^ 5X buffer and a primer final concentration of 0.3 μM in a 30-μl reaction volume. The reactants were initially denatured at 95°C for 1 min, followed by 35 cycles of 95°C for 15 s, 61°C for 15 s, 72°C for 10 s, and a final extension at 72°C for 5 min. During the selection of transgenic plants it was assumed that the OE-*OsNAS* constructs had a dominant effect despite of the insert zygocity. Forty eight OE-*OsNAS*/IR64 and OE-*OsNAS*/Esp transgenic plants were used in a completely randomized block design with three replicated blocks for each progeny. Null segregants were cultivated in independent culture boxes.

### Agro-morphological Characterization

Thirteen agro-morphological traits were evaluated at flowering stage: (1) days to 50% flowering; and at maturity including (2) culm number; (3) plant height; (4) panicle length; (5) number of filled grain per main panicle; (6) total number of grain per main panicle; (7) spikelet fertility; (8) estimated grain yield per plant – calculated by multiplying the number of filled grain per panicle by the number of productive culms and dry weight (DW) per grain; (9) root DW; (10) stem/sheath DW; (11) non-flag leaf DW; (12) flag leaf DW; and (13) panicle DW (panicle stalk and pedicels only).

### Elemental Analysis of Plant Tissue

At maturity, plant tissue was dissected into roots, stems/sheaths, non-flag leaves, flag leaves, panicles (panicle stalk and pedicels only) and grain (caryopsis only). Vegetative tissue was submerged in an ultrapure water bath (18.2 Ω) for 10 s to reduce Fe and Zn contamination. Plant material was oven-dried at 60°C for 72 h and ground to a fine powder using a non-contaminating zirconium jar (MEP instruments Pty Ltd, NSW, Australia) and a Qiagen Retsch MM300 TissueLyser. Polished grain was produced using a modified commercial bench-top miller (Kett Electrical Laboratory, Tokyo, Japan) with a polishing time of 30 s. Polished grain samples were washed for 10 s with ultrapure water (18.2 Ω) and then dried for 10 min at 37°C before elemental analyses. Elemental concentrations of ground tissue and grain samples were determined by ICP-OES at Waite Analytical Services (Adelaide, SA, Australia) (Wheal et al., 2011). Total Fe and Zn contents in individual plant tissues were calculated by multiplying Fe and Zn concentrations by dry weight. Percentages of Fe and Zn in polished grain were calculated by multiplying polished grain concentration by 100 and dividing by unpolished grain concentration.

### Statistical Analysis

Significant differences between transgenic plants and NS were determined by one-way ANOVA (*P* < 0.05) followed by *post hoc* tests (*P* ≤ 0.05) Hochberg’s GT2 (when progeny sample numbers were unbalanced) or Fisher’s least significant difference (LSD; when progeny sample number were balanced). Normality and homoscedasticity assumptions were tested using Shapiro-Wilk and Levene’s tests (*P* < 0.05), respectively. Data was transformed using natural logarithm when normality and homoscedasticity assumptions were violated. Percentages were transformed using Arcsin [sqrt (spikelet fertility/100)]. Analyses were performed using the IBM SPSS Base 23.0 for PC (SPSS, IBM).

A distance-based linear model (DistLM) was implemented to determine which plant tissue nutrient predictors contributed to the variation in the agro-morphological traits. A total of 48 predictors were included in the DistLM analyses, and were obtained from eight nutrients assessed in six plant tissues (Supplementary Table 1). The biological dataset for each progeny was built from 9 to 4 agro-morphological traits for the OE-*OsNAS*/IR64 and OE-*OsNAS*/Esp progenies, respectively (Supplementary Table 2). A stepwise routine was run employing 999 permutations and using the AICc (Akaike’s information criterion corrected) selection criterion on a log-transformed matrix of predictors, and a resemblance matrix of agro-morphological traits based on Euclidian distance. Distance-based redundancy analysis (dbRDA) was used for graphical representation of the DistLM results (Legendre and Anderson, 1999). The predictors were superimposed onto the dbRDA plot as vectors whose direction and length are related to their partial correlation with the dbRDA axes, and allow visualizing the role they played in generating the ordination of individual plant phenotypes in the dimensional space. Multivariate statistical analyses were performed using PRIMER V6 statistical package with the PERMANOVA+ add-on (PRIMER-E, Plymouth Marine Laboratory, UK).

## Results

### Iron and Zn Concentrations in Unpolished Grain and Association with Grain Yield

**FIGURE 1:**
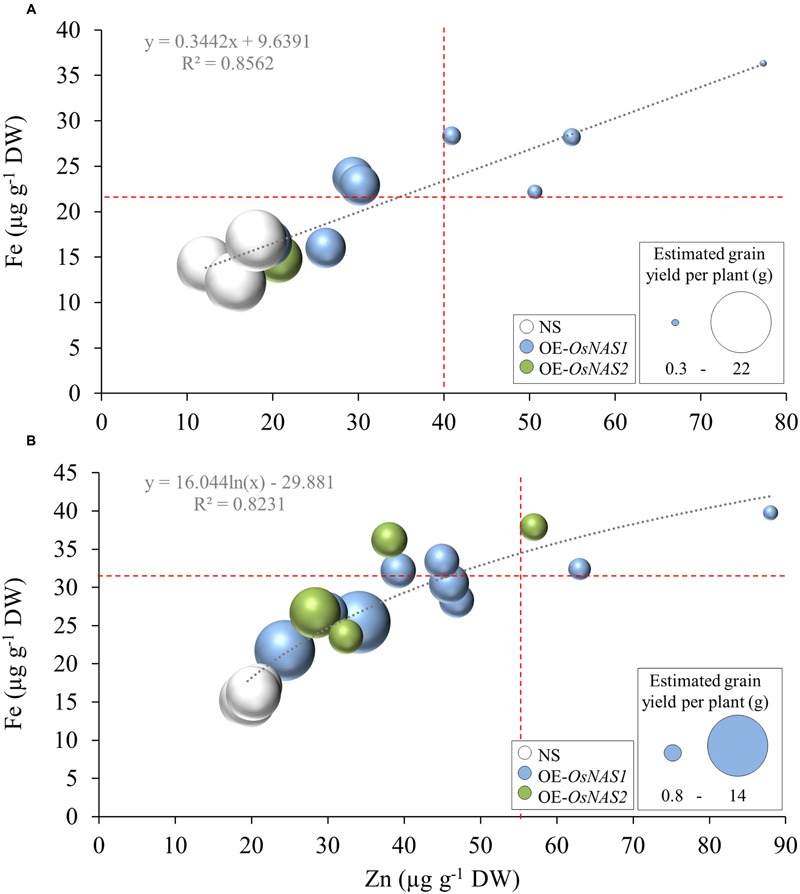
**Variation in grain Fe and Zn concentrations and association with grain yield in the **(A)** OE-*OsNAS*/IR64 and **(B)** OE-*OsNAS*/Esp progeny.** Different sizes in bubbles represent variation in estimated grain yield per plant. Red dotted lines indicate Fe and Zn concentrations from which estimated grain yield per plant was reduced.

Overall, the transgenic BC_2_F_2_ progeny contained up to 148% higher Fe and 336% higher Zn concentrations in unpolished grain compared to the NS progeny. The largest increase percentage in grain Fe and Zn concentrations were observed in the OE-*OsNAS*/Esp progeny (**Figures 1A,B**). Variation in grain Fe concentration in the transgenic progeny ranged from 14 to 40 μg g^-1^ DW whereas variation in grain Zn concentration ranged from 14 to 88 μg g^-1^ DW. Unpolished grain concentrations surpassing 23 μg Fe g^-1^ and 40 μg Zn g^-1^ in the OE-*OsNAS*/IR64 progeny, and 36 μg Fe g^-1^ and 56 μg Zn g^1^ in the OE-*OsNAS*/Esp progeny, were associated with significant reductions in grain yield. There was no association between the lowest grain yield values and the OE-*OsNAS* construct (**Figures 1A,B**). Furthermore, there was no association between *OsNAS* expression levels and Fe/Zn concentrations in unpolished rice grain (Supplementary Figure 2). Ranges of variation in number of filled grain per main panicle, spikelet fertility and estimated grain yield per plant relative to the NS progeny, were used to classify transgenic (+) progeny as high-yield (+HY) or low-yield (+LY) (**Table 1**). The OE-*OsNAS*/IR64 +LY progeny included four plants containing the OE-*OsNAS1* construct, whereas the OE-*OsNAS*/Esp +LY progeny included two plants containing the OE-*OsNAS1* construct and one containing the OE-*OsNAS2* construct (**Figures 1A,B**).

**Table 1:** Variation in grain yield components used to classify transgenic progeny into high-yield (+HY) and low-yield (+LY).

Grain yield component	Range of variation (Minimum–Maximum)					
	OE-*OsNAS*/IR64			OE-*OsNAS*/Esp		
	NS	+HY	+LY	NS	+HY	+LY
Number of filled grain per panicle	100–126	83–116	4–35	124–127	55–150	13–45
Spikelet fertility (%)	67–82	71–89	5–39	7–89	70–96	14–36
Estimated grain yield per plant (g)	19–22	9–14	0.3–2	8–11	4–14	0.8–4

### Agro-morphological Traits of High and Low-Yield Transgenic Progeny

The yield components listed in **Table 1** were all significantly reduced in the +LY crosses of both crosses compared to the +HY and NS progenies. Additionally, the OE-*OsNAS*/IR64 +LY progeny had significantly reduced plant height and dry weight of the root, stem/sheath and panicle, whereas flowering was significantly delayed in the OE-*OsNAS*/Esp +LY progeny (**Table 2**). The OE-*OsNAS*/IR64 +HY progeny also showed a reduction in culm number, total grain per main panicle and estimated grain yield per plant compared to the NS progeny. However, fertility of the OE-*OsNAS*/IR64 +HY progeny was not affected (**Table 2**). By contrast, the OE-*OsNAS*/Esp +HY progeny showed no significant differences for all of the agro-morphological traits compared to the NS progeny (**Table 2**). No significant differences were observed in any agro-morphological traits between transgenic progeny containing the OE-*OsNAS1* or OE-*OsNAS2* constructs (Supplementary Table 3).

**Table 2:** Agro-morphological performance of transgenic progeny classified as high-yield (+HY) and low-yield (+LY) in the OE-*OsNAS*/IR64 and OE-*OsNAS*/Esp progenies.

Progeny type	Days to 50% flowering	Culm No	Plant height (cm)	Panicle length (cm)	Number of filled grain per main panicle	Total grain per main panicle	Spikelet fertility (%)	Estimated grain yield per plant (g)	Dry weight (g)				
									Root	Stem/sheath	Non-flag leaf	Flag leaf	Panicle
*OE-OsNAS/IR64*													
NS (n = 3)	99	11^a^	92.2^a^	24.2	110^a^	153^a^	72.22^a^	20.90^a^	3.80^a^	17.07^a^	5.13	1.18	0.65^a^
+HY (*n* = 6)	100	7^b^	87.6^a^	22	93^a^	115^b^	80.14^a^	11.39^b^	2.74^ab^	11.74^ab^	3.76	1.02	0.46^ab^
+LY (*n* = 4)	103	5^b^	63.9^b^	25.4	19^b^	93^b^	20.64^b^	1.32^c^	1.42^b^	5.50^b^	2.76	0.64	0.18^b^
*P*-value _(progenytype)_	n.s.	^∗∗^	^∗∗^	n.s.	^∗∗∗^	^∗∗^	^∗∗∗^	^∗∗∗^	^∗∗^	^∗∗^	n.s.	n.s.	^∗∗^

*OE-OsNAS/Esp*													
NS (*n* = 3)	94^b^	4	102	22.8	126^a^	140	88.39^a^	9.97^a^	1.58	7	2.47	0.52	0.28
+HY (*n* = 10)	98^ab^	4	93.1	20.4	86^a^	103	83.60^a^	7.07^ab^	1.76	5.77	2.41	0.44	0.21
+LY (*n* = 3)	102^a^	3	95.6	22.5	28^b^	108	25.12^b^	2.15^b^	2.11	7.12	2.41	0.52	0.2
*P*-value _(progenytype)_	^∗^	n.s.	n.s.	n.s.	^∗∗^	n.s.	^∗∗∗^	^∗^	n.s.	n.s.	n.s.	n.s.	n.s.

n.s., Not significant. Within each column, values with different letters represent significant differences between progeny type at the 5% level by Hochberg’s GT2 test. Values given are means. *P < 0.05, **P < 0.01, ***P < 0.001. NS, null segregants.

### Fe and Zn Accumulation in Different Plant Tissues of High and Low-Yield Transgenic Progeny

Differences in Fe and Zn concentrations of flag leaf, panicle, and grain were observed between the OE-*OsNAS*/IR64 +LY progeny and the NS progeny. Zinc concentrations in the flag-leaf of the OE-*OsNAS*/IR64 +LY progeny were 69% higher, while Fe and Zn concentrations in the panicle were 37% and 105% higher, relative to the NS progeny. In grain of the OE-*OsNAS*/IR64 +LY progeny, Fe and Zn concentrations were 100 and 265% higher than the NS progeny (**Table 3**). No significant differences between the OE-*OsNAS*/IR64 +HY and the NS progeny were observed regarding the Fe and Zn concentrations of any plant tissue. In the OE-*OsNAS*/Esp progeny, increases in Fe and Zn concentrations were only observed in the grain of both +LY and +HY progenies. Grain Fe and Zn concentrations in the OE-*OsNAS*/Esp +LY were 124 and 212% higher than the NS progeny, whereas grain Fe and Zn concentrations in the OE-*OsNAS*/Esp +HY were 78 and 90% higher than the NS grain (**Table 3**).

**Table 3:** Iron and Zn concentrations in individual plant tissues of transgenic progeny classified as high-yield (+HY) and low-yield (+LY) in the OE-*OsNAS*/IR64 and OE-*OsNAS*/Esp progenies.

Progeny type	Concentration (μg g^-1^ DW)											
	Root		Stem/sheath		Non-flag leaf		Flag leaf		Panicle		Grain	
	Fe	Zn	Fe	Zn	Fe	Zn	Fe	Zn	Fe	Zn	Fe	Zn
*OE-OsNAS/IR64*												
NS (*n* = 3)	6267	18.7	273	7.8	237	10.2	205	9.8^b^	89^b^	16.9^b^	14.4^b^	15.3^b^
+ HY (*n* = 6)	7350	18.5	258	12.0	251	10.6	213	11.3^b^	107^ab^	11.1^b^	18.0^b^	23.2^b^
+ LY (*n* = 4)	9150	22.5	283	26.2	253	15.6	193	16.6^a^	122^a^	34.6^a^	28.8^a^	55.9^a^
*P*-value _(progenytype)_	n.s.	n.s.	n.s.	n.s.	n.s.	n.s.	n.s.	^∗∗^	^∗^	^∗∗^	^∗∗^	^∗∗∗^
*OE-OsNAS/Esp*												
NS (*n* = 3)	8000	25.0	207	9.0	295	13.2	295	15.2	155	10.0	16.1^b^	20.2^b^
+ HY (n = 10)	8120	43.4	234	19.0	337	13.7	296	17.9	157	15.2	28.6^a^	38.3^a^
+ LY (*n* = 3)	6333	59.0	243	16.0	423	10.5	320	14.2	173	16.1	36.1^a^	63.0^a^
*P*-value _(progenytype)_	n.s.	n.s.	n.s.	n.s.	n.s.	n.s.	n.s.	n.s.	n.s.	n.s.	^∗∗∗^	^∗∗∗^

n.s., not significant. Within each column, values with different letters represent significant differences between progeny type at the 5% level by Hochberg’s GT2 test. The values given are means. *P < 0.05, **P < 0.01, ***P < 0.001. NS, null segregants; DW, dry weight.

**FIGURE 2:**
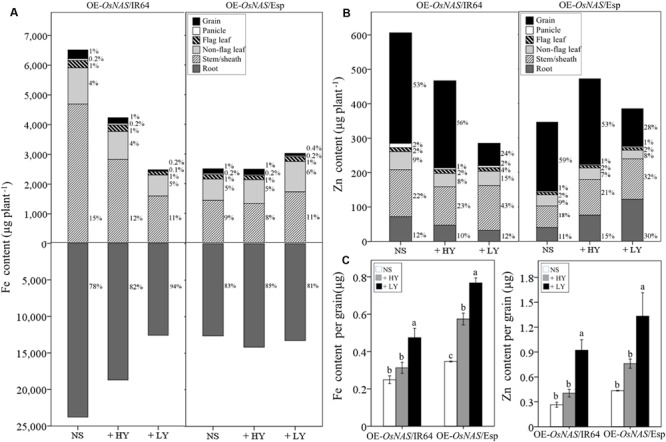
**Fe and Zn allocation to different plant tissues of transgenic plants classified as high yield (+HY) and low yield (+LY) in the OE-*OsNAS*/IR64 and OE-*OsNAS*/Esp progenies.** Stacked bar graphs represent **(A)** Fe content and **(B)** Zn content in individual plant tissues. **(C)** Fe and Zn content per grain. % values indicate the percentage of Fe or Zn allocation to each tissue within a bar. Error bars are ±SE. The values given are means of *n* = 3–10. Grain = caryopsis only.

Total plant Fe and Zn contents were reduced in the OE-*OsNAS*/IR64 +LY progeny compared to the NS progeny (**Figures 2A,B**). However, this was a consequence of significant reductions in dry weight rather than reductions in Fe and Zn concentrations (**Tables 2** and **3**). By contrast, total plant Fe and Zn contents of the OE-*OsNAS*/Esp +HY and +LY tended to increase compared to the NS progeny (**Figures 2A,B**), suggesting increased uptake of Fe and Zn by *OsNAS* overexpression. We calculated percentages of Fe and Zn allocations to different plant tissues to identify shifts of Fe and Zn accumulation at maturity in the transgenic progeny. Allocation of Fe and Zn to hulls was not considered. The percentage of Fe allocation to the grain was lower in the +LY progenies compared to the +HY and NS progenies (**Figure 2A**). The +LY progenies had the highest percentage of Zn allocation to the stem/sheath whereas the +HY and NS progenies had the highest percentage of Zn allocation to the grain (**Figure 2B**). Additionally, the percentage of Zn allocation to the non-flag leaf and flag leaf of the OE-*OsNAS*/IR64 +LY progeny was higher than that of the OE-*OsNAS*/IR64 +HY and NS progenies (**Figure 2B**). Iron and Zn contents per grain were increased in the OE-*OsNAS*/IR64 +LY, and in the OE-*OsNAS*/Esp +LY and +HY progenies (**Figure 2C**). In the OE-*OsNAS*/IR64 +LY progeny, Fe and Zn contents per grain were 91% (*P* = 0.007) and 247% (*P* < 0.001) higher than the NS progeny. In the OE-*OsNAS*/Esp +LY progeny, Fe and Zn contents per grain were 121% (*P* < 0.001) and 206% higher (*P* < 0.01) than the NS (**Figure 2C**). Moreover, Fe content per grain in the OE-*OsNAS*/Esp +HY was 66% higher than the NS (**Figure 2C**).

### Plant Tissue Nutrient Predictors Accounting for the Largest Reductions in Agro-morphological Traits of Low-Yield Transgenic Progeny

The DistLM analyses identified three and four predictors in the OE-*OsNAS*/IR64 and OE-*OsNAS*/Esp progeny, respectively, which best explained the variation in the selected agro-morphological traits (**Table 4**). In the OE-*OsNAS*/IR64 progeny, the three predictors were grain-Zn, panicle-Mg and non-flag leaf-P and together they explained 89.4% of the variation in nine agro-morphological traits showing significant differences between the OE-*OsNAS*/IR64 +LY progeny and the NS progeny (**Table 2**). In the OE-*OsNAS*/Esp progeny, the four extracted predictors were panicle-Mg, grain-Zn, stem/sheath-Zn and non-flag leaf-K, and together they explained 91.7% of the variation in four agro-morphological traits showing significant differences between the OE-*OsNAS*/Esp +LY progeny and the NS progeny (**Table 2**).

**Table 4:** Distance-based linear model of agro-morphological traits against 48 plant tissue nutrient predictors.

Marginal tests			Sequential tests			
Predictor	Pseudo-F	% Variance explained	Cumulative AICc	Pseudo-F	% Variance explained	% Cumulative variance
*OE-OsNAS/IR64*						
Grain-Zn	21.7^∗∗∗^	66.4	7.1	21.7^∗∗∗^	66.4	66.4
Panicle-Mg	1.3^n.s.^	10.4	0.7	11.3^∗∗∗^	17.9	84.2
Non-flag leaf-P	3.0^n.s.^	21.7	-0.1	4.4^∗∗^	5.2	89.4
*OE-OsNAS/Esp*						
Panicle-Mg	24.7^∗∗∗^	63.8	-4.7	24.7^∗∗∗^	63.8	63.8
Grain-Zn	11.6^∗∗^	45.3	-10.7	10.0^∗∗∗^	15.7	79.5
Stem/sheath-Zn	2.2^n.s.^	13.5	-16.9	10.1^∗∗^	9.4	88.9
Non-flag leaf-K	1.8^n.s.^	11.1	-17.2	3.8^∗∗^	2.8	91.7

*n.s., Not significant. Proportion of variance in 4 and 9 agro-morphological traits for the OE-*OsNAS*/IR64 and OE-*OsNAS*/Esp progenies, respectively, is explained by the best extracted predictors in stepwise sequential tests following AIC_*c*_ selection criterion. Marginal tests from DistLM are also presented. ^∗∗^*P* < 0.01, ^∗∗∗^*P* < 0.001.*

**FIGURE 3:**
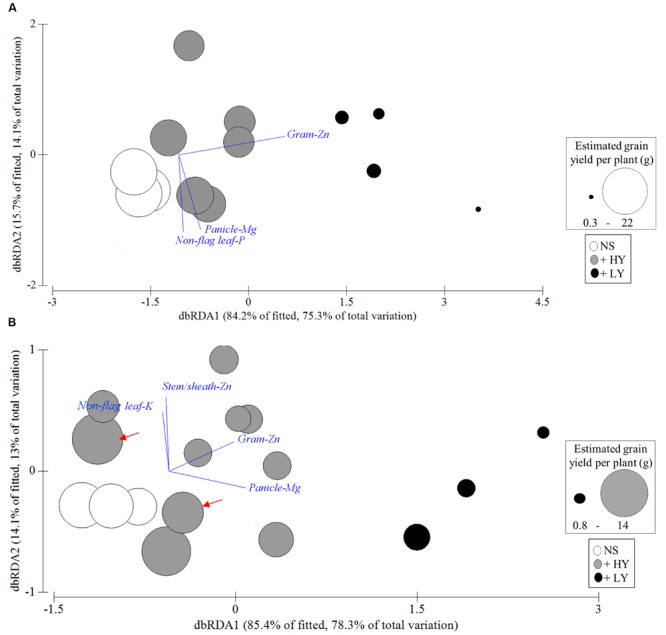
**Distance-based redundancy analysis (dbRDA) illustrating the Distance-based linear model (DistLM) for the **(A)** OE-*OsNAS*/IR64 and **(B)** OE-*OsNAS*/Esp progeny.** The best predictors explaining the largest percentage of the variation in the agro-morphological traits are superimposed onto the dbRDA biplot. Their direction and length are related to their partial correlation with the dbRDA axes. Predictors correlated with the dbRDA1 accounted for the largest plant grain yield reductions. Different sizes in bubbles represent variation in estimated grain yield per plant. NS, null segregant; +HY, high-yield transgenic progeny; +LY, low-yield transgenic progeny. Data represents individual replications. The OE-*OsNAS*/Esp +HY progeny used for polished rice analyses is pointed with the red arrows.

Graphical representation of the DistLM analyses using dbRDA is presented in **Figure 3**. Overall, the predictors correlated with the dbRDA1 axis had a larger effect on the spatial separation due to dissimilarities between the +LY, and the +HY and NS progenies, whereas predictors correlated with the dbRDA2 axis had a larger effect on the spatial separation due to dissimilarities attributed to variation among transgenic progenies (**Figures 3A,B**).

In the OE-*OsNAS*/IR64 progeny, the predictor grain-Zn showed the largest positive multiple partial correlation with the dbRDA1 axis (*r* = 0.979) and explained 66.4% of total variation in the agro-morphological traits (**Table 4**). This was reflected by the large correlation given by the length and direction of the grain-Zn vector in the dbRDA1 that explained 75.3% of the total variation (**Figure 3A**). The contribution of the predictor grain-Zn to variability in the agro-morphological traits of the OE-*OsNAS*/IR64 progeny was the largest and this was the only statistically significant predictor based on the DistLM marginal tests (**Table 4**). The predictor panicle-Mg was the second most influential predictor explaining an additional 17.9% to the total variation in agro-morphological traits (**Table 4**). This predictor showed a negative correlation with the dbRDA2 axis (*r* = -0.684) and its contribution was significant for sequential tests but not for the marginal test (**Table 4**). The predictor non-flag leaf-P explained an additional 5.2% of the total variation in the agro-morphological traits in the sequential tests but its contribution in the marginal tests was not significant (**Table 4**). These results demonstrate that grain-Zn was the primary predictor positively associated with the largest grain yield reductions in the OE-*OsNAS*/IR64 progeny.

In the OE-*OsNAS*/Esp progeny, the predictors panicle-Mg and grain-Zn had the largest positive multiple partial correlation with the dbRDA1 axis (*r* = 0.765 and *r* = 0.640), contributing to 63.8% and 15.7% of the total variation in the agro-morphological traits, respectively (**Table 4**). These two predictors were associated with the OE-*OsNAS*/Esp +LY progeny in the dbRDA1 axis which accounted for 78.3% of the total variation (**Figure 3B**). Individual contributions of the predictors panicle-Mg and grain-Zn to the variation in the agro-morphological traits was significant (marginal tests, **Table 4**). The predictors stem/sheath-Zn and non-flag leaf-K both explained an additional 12.2% in sequential tests but their individual contribution was not significant (**Table 4**). These results suggest that panicle-Mg and grain-Zn were the primary predictors positively associated with the largest grain yield reductions in the OE-*OsNAS*/Esp progeny.

### Polished Grain Fe and Zn Concentrations of High-Yield Transgenic Progeny Containing OE-*OsNAS1* or OE-*OsNAS2* Constructs

**FIGURE 4:**
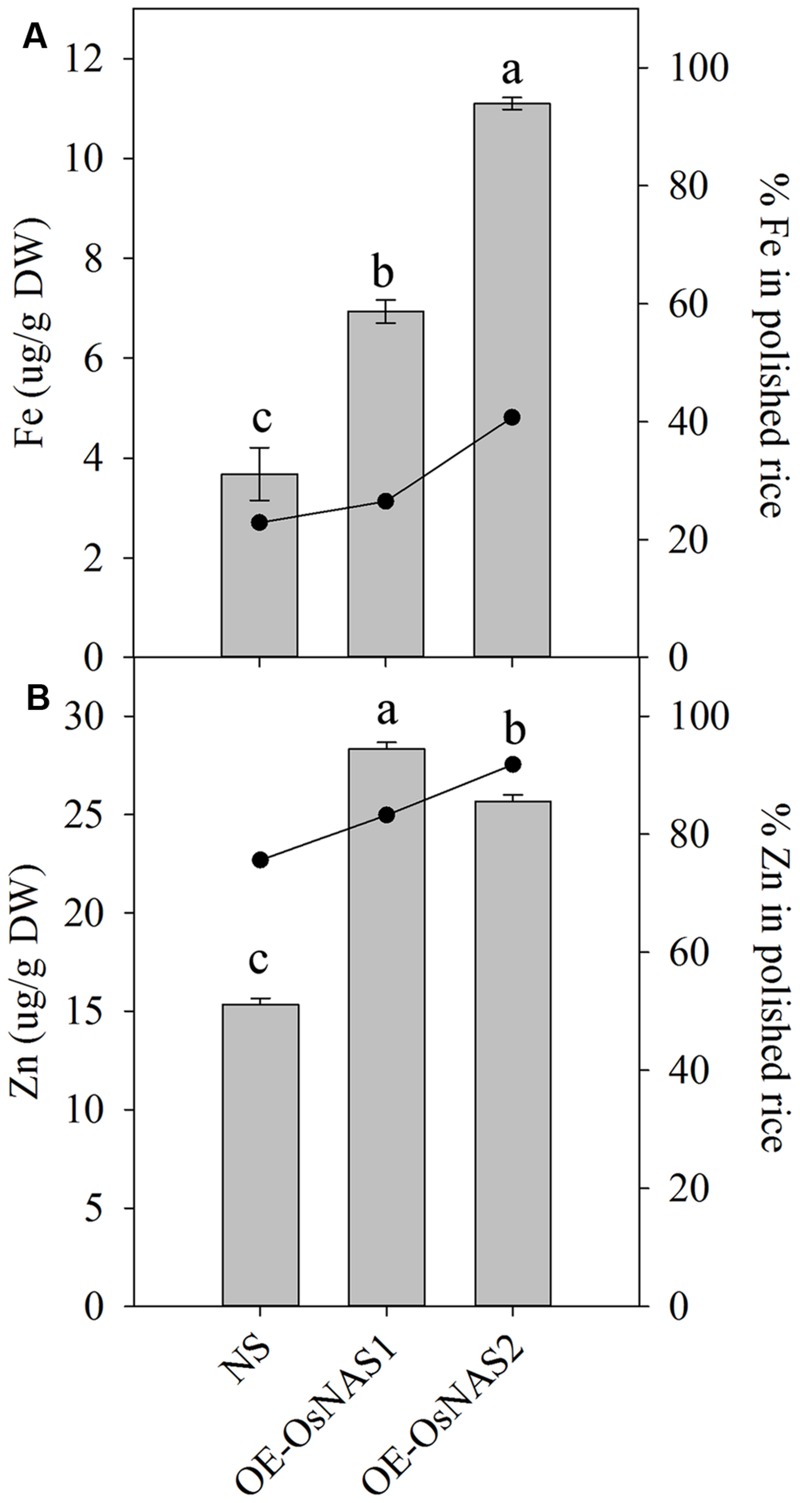
**Iron and Zn concentrations (gray solid bars) and percentages (black line) in polished grain of selected OE-*OsNAS*/Esp high-yield transgenic progeny containing either the OE*-OsNAS1* or OE*-OsNAS2* constructs. (A)** Fe concentration and percentage in polished grain. **(B)** Zn concentration and percentage in polished grain. Letters represent statistical differences at the 5% level by Fisher’s LSD test for mineral concentration. Bars represent mean values of *n* = 3. Error bars are ±SE. NS, null segregants.

OE-*OsNAS*/Esp +HY progeny harboring either the OE-*OsNAS1* or OE-*OsNAS2* constructs were selected based on similar Fe concentrations in unpolished rice grain of 26 and 27 μg Fe g^-1^ DW, respectively (**Figure 3**, red arrows). Concentrations of Zn in unpolished rice grain were in the lowest 15% of concentration of grain of the OE-*OsNAS*/Esp progeny with 34 μg Zn g^-1^ DW and 28 μg Zn g^-1^ DW for the plants containing the OE-*OsNAS1* and OE-*OsNAS2* construct, respectively. We did not identify high-Fe OE-*OsNAS*/IR64 +HY progeny harboring the OE-*OsNAS2* construct from the small number of BC_2_F_2_ progeny used for mineral assessment in this study. Progeny harboring the OE-*OsNAS1* and OE-*OsNAS2* constructs showed significant increases in Fe and Zn concentrations compared to the NS progeny (**Figures 4A,B**). Iron concentrations in polished rice of OE-*OsNAS1* and OE-*OsNAS2* plants was 7 and 11 μg Fe g^-1^ DW, representing 86 and 197% higher Fe, respectively, compared to the NS progeny (**Figure 4A**). Zinc concentrations in polished grain of plants containing the OE-*OsNAS1* and OE-*OsNAS2* constructs were 28 and 25 μg Zn g^-1^ DW, representing 85 and 68% higher Zn concentrations, respectively, compared to the NS progeny (**Figure 4B**). Importantly, progeny containing the OE-*OsNAS2* construct showed a higher percentage of Fe and Zn in polished rice compared to plants harboring the OE-*OsNAS1* construct (**Figures 4A,B**). These results indicate that the OE-*OsNAS2* construct induces higher translocation of Fe and Zn into deeper layers of the outer endosperm which are not lost during the process of polishing.

## Discussion

### Variation in Grain Fe and Zn Concentrations of Transgenic Progeny Was Associated with Variations in Grain Yield

BC_2_F_2_ progeny containing OE-*OsNAS1* and OE-*OsNAS2* constructs contained up to 40 μg Fe g^-1^ DW and 88 μg Zn g^-1^ DW in unpolished rice grain, representing 148 and 336% higher Fe and Zn concentrations, respectively, compared to NS progeny. Although there was a strong and positive correlation between grain Fe and Zn concentrations, the variation in grain Fe concentrations was smaller than the variation in grain Zn concentrations in transgenic progeny (**Figure 1**). This result could be explained by differences in remobilization efficiency of Fe and Zn to the grain. In rice phloem sap, the major ligand of Zn is NA whereas Fe is predominantly bound to DMA (Nishiyama et al., 2012), indicating that NA overproduction may have a more pronounced effect on increasing Zn remobilization (via phloem) to the grain.

Unpolished grain concentrations surpassing 23 μg Fe g^-1^ and 40 μg Zn g^-1^ in the OE-*OsNAS*/IR64 progeny, and 38 μg Fe g^-1^ and 56 μg Zn g^-1^ in OE-*OsNAS*/Esp progeny, were associated with significant reductions in grain yield. Likewise, plants with the lowest grain Fe and Zn concentrations showed some of the highest grain yield values (**Figure 1**). The negative association between grain yield and micronutrient concentrations can be explained by important source-sink relationships within the plant. Nutrient concentrations in plant tissues tend to decrease as dry matter production increases, a phenomenon often described as “dilution” (Rengel, 2002) and which explains the low concentration of grain Fe and Zn associated with the highest grain yield values. Dilution of micronutrients in grain can also be due to faster growth rate of the grain relative to the rate of micronutrient loading (Rengel, 2002). Rice genotypes with faster rates of Zn loading often have higher grain Zn concentrations (Wu et al., 2010). The higher grain Fe and Zn concentrations of the +LY and +HY progenies in this study may be due to faster rates of Fe and Zn loading compared to growth rate of the grain, but this requires further investigation. Grain number is an important trait determining grain nutrient concentrations in rice (Sperotto et al., 2013). By removing 50% of grain from rice panicles at anthesis, Sperotto et al. (2013) reported significant increases in Fe and Zn concentrations in the remaining grain suggesting that fixed amounts of Fe and Zn are translocated to the panicle independently of grain number. Number of filled grain per main panicle was drastically reduced in the +LY progenies (**Table 2**), thus low number of grain was a major factor enhancing grain Fe and Zn concentrations in those progenies. However, this was not the case for the OE-*OsNAS*/Esp +HY progeny which showed significantly higher concentrations of Fe and Zn in the grain compared to the NS with no reduction in the number of grain per main panicle (**Tables 2** and **3**). Taken all the above together, we conclude that micronutrient dilution and number of grain are major factors influencing the lowest and highest grain Fe and Zn concentrations in the progenies of the current study. However, significantly higher concentrations of Fe and Zn can be achieved by *OsNAS* overexpression without any reduction in grain number, suggesting that it is feasible to couple high grain Fe and Zn concentrations with high grain yields in biofortified rice.

### Grain-Zn and Panicle-Mg Were the Primary Predictors Associated with Low-Yield Progeny

In the current study, grain-Zn accounted for 66% of total variance in agro-morphological traits of the OE-*OsNAS*/IR64 progeny, whereas panicle-Mg accounted for 64% of the total variance in agro-morphological traits of the OE-*OsNAS*/Esp progeny (**Table 4**). The high variance percentages accounted by just one individual predictor in each genetic background is unexpected considering the large number of predictors involved in the multivariate analyses. A number of studies have reported increased concentrations of Zn in rice roots, shoots and leaf tissues by *OsNAS1* and *OsNAS2* overexpression (Lee et al., 2009, 2011, 2012; Zheng et al., 2010) and Lee et al., 2012 reported overaccumulation of Zn in vegetative tissues to be associated with reduced grain yield. However, leaf Zn concentrations in those studies, as well as leaf Zn concentrations of +LY progenies in the current study, did not reach Zn toxicity levels which are reported to range from 100 to 700 μg g^-1^ DW in most plants (Fageria, 2016). This result suggests that the association of high grain-Zn with reduced yield in +LY progenies was not due to Zn toxicity in vegetative tissues and that high Zn may have impacted on other physiological processes related to reproduction and grain fill. In developing tissues such as meristems and reproductive organs, Zn is required in high levels as it is involved in many enzyme activities and cell developmental processes including metabolism of auxin (Broadley et al., 2012). For instance, high Zn concentrations at the growing tips of pollen tubes enable rapid cell division (Ender et al., 1983). However, excessively high Zn concentrations can interfere in DNA replication and in turn impair the development of reproductive cells (MacDonald, 2000). Comparative analysis of Zn concentrations in reproductive organs of the +HY and +LY progenies could help determine if there are detrimental effects of Zn overaccumulation on reproductive development and/or grain fill. The identification of panicle-Mg as a major predictor of grain yield variation in the *OsNAS*/Esp progeny was unexpected as there is no evidence indicating that NA or DMA can chelate or mobilize this macronutrient. The OE-*OsNAS*/Esp +LY progeny with the highest grain Fe and Zn concentrations also had the highest panicle Mg concentrations, suggesting that high panicle-Mg may be an indirect consequence of the highest Fe and Zn concentrations in the grain in this genetic background (Supplementary Figure 4). The large association of panicles-Mg with +LY progenies, particularly for the +LY OE-*OsNAS*/Esp progeny, requires further investigation.

Increased activity of the *OsNAS1* and *OsNAS2* transgenes in the OE-*OsNAS* progeny, leading to increased NA and DMA concentrations within the plant, is an important factor that could also affect grain yield. Results from our group have identified that NA and DMA concentrations in xylem sap of selected OE-*OsNAS*/IR64+LY progeny contained only 130% higher NA and 170% higher DMA compared to the NS progeny (Supplementary Figure 3). Likewise, there was no correlation between transcriptional activity of the *OsNAS* genes in selected OE-*OsNAS*/IR64 progeny with grain Fe and Zn concentrations or grain yield (Supplementary Figure 2). These results indicate that increased activity of the *OsNAS1* and *OsNAS2* transgenes in the OE-*OsNAS* progeny populations does not appear to be responsible for the reduced yield of +LY plants.

### Genetic Background Has a Major Effect on the Development of Fe and Zn Biofortified Rice without Yield Penalty

Whole-plant mineral assessment of the OE-*OsNAS*/Esp +HY and +LY progenies show that *OsNAS* overexpression clearly increases Fe and Zn uptake (**Figures 2A,B**). The results also show that *OsNAS* overexpression increases Fe and Zn allocation to the grain as observed by the increased grain Fe and Zn contents of the +HY progenies (**Figures 2A,B**). However, genotype-dependent physiological limits for Fe and Zn accumulation may exist within plant tissues and grain. Grain yield per plant in the *japonica/japonica* OE-*OsNAS*/Esp +LY progeny was reduced by 78% whereas in the *indica/indica* OE-O*sNAS*/IR64 +LY progeny grain yield was reduced by 94% (**Table 3**). Moreover, reductions in dry weight of vegetative tissue were not observed in the OE-*OsNAS*/Esp +LY progeny compared to the OE-O*sNAS*/IR64 +LY progeny (**Table 3**) despite accumulating 0.2 μg more Fe and 0.4 μg more Zn per grain (**Figure 2C**). Differences in phenotypic performance between the two genetic backgrounds are likely influenced by genetic variation in grain Fe and Zn concentrations associated with uptake and remobilization of these micronutrients. For instance, under sufficient Fe nutrient environments, Fe concentrations in mature upper leaves and flag leaves of *japonica* rice continue to increase during late stages of development, suggesting that these tissues serve as important sources for grain Fe loading (Yoneyama et al., 2010; Sperotto et al., 2012b). Accordingly, the higher concentrations of Fe in the non-flag leaf and flag-leaf of the *japonica/japonica* genetic background compared to the *indica/japonica* background (**Table 3**) may have provided an increased pool of Fe for higher remobilization to the grain by *OsNAS* overexpression. Increased uptake during grain filling, however, may have also served as an important source for grain Fe and Zn in the *japonica/japonica* genetic background, as total Fe and Zn contents tended to increase in the OE-*OsNAS*/Esp +HY and +LY progenies compared to the NS progeny (**Figures 2A,B**). In rice, Zn re-allocation from stems is an important source for Zn remobilization to reproductive tissues (Jiang et al., 2008; Wu et al., 2010), and is considered a major barrier to enhanced Zn allocation toward the grain (Stomph et al., 2014). Compared to the *japonica/japonica* genetic background, the *indica/japonica* background had a higher percentage allocation of Zn to the stem/sheath (**Figure 2B**), which suggests that the *indica/japonica* genetic background may be less efficient at allocating Zn (and probably Fe) to the grain. However, since Zn concentrations in leaves and panicles of the OE-*OsNAS*/IR64 +LY progeny were significantly higher compared to the NS progeny (**Table 3**), increased Zn uptake is likely promoted by *OsNAS* overexpression in the *indica/japonica* background, and may be responsible for the reductions in dry weight of leaves and panicles (**Table 2**). Taken together, these results suggest that the *japonica/japonica* background of the OE-*OsNAS*/Esp progeny may be better suited to tolerate the large Fe and Zn increases conferred by *OsNAS* overexpression.

### High-Yield Progeny Containing *OsNAS2* Overexpression Constructs Showed Higher Percentage of Fe and Zn in the Polished Rice Grain

High-yield OE-*OsNAS*/Esp progeny containing the OE-*OsNAS2* construct had 11 μg Fe g^-1^ DW and 26 μg Zn g^-1^ DW in polished grain and showed a higher percentage of Fe and Zn in polished grain compared to the OE-*OsNAS*/Esp progeny containing the OE-*OsNAS1* construct (**Figures 4A,B**), thus supporting the findings in Johnson et al. (2011). Rice shows higher percentages of Fe loss during polishing compared to Zn because Fe concentrations in rice are highest in the bran of the rice grain, and in the scutellum tissue inside the germ layer which are lost during polishing, whereas Zn is preferentially accumulated in the central endosperm tissues of the grain (Lu et al., 2013). In the current study, overexpression of both *OsNAS1* and *OsNAS2* was efficient at increasing concentrations of Fe and Zn in unpolished grain compared to the NS. However, higher percentages of Fe and Zn in polished grain from OE-*OsNAS*/Esp progeny containing the OE-*OsNAS2* construct compared to progeny containing the OE-*OsNAS1* construct indicates that *OsNAS2* overexpression leads to higher translocation of Fe and Zn into the endosperm, thus providing a more efficient strategy to develop Fe and Zn biofortified polished rice.

Finally it is important to mention that the grain Zn overaccumulation thresholds associated with low yield in the current study, a growth room-based hydroponics experiment, may differ from those in a field setting. Thus, multi-location field evaluations of advanced OE-*OsNAS*/IR64 and the OE-*OsNAS/*Esp backcross progenies are necessary to verify that these results apply to the growth and yield of Fe and Zn biofortified rice under field conditions.

## Conclusion

This study reported for the first time agro-morphological performance and nutrient accumulation at the whole-plant level for BC_2_F_2_ progeny containing OE-*OsNAS1* and OE-*OsNAS2* constructs under *indica/japonica* and *japonica/japonica* genetic backgrounds. We identified up to 148% higher Fe and 336% higher Zn concentrations in unpolished rice relative to the NS progeny. However, in a hydroponic environment, unpolished rice concentrations surpassing 23 μg Fe g^-1^ and 40 μg Zn g^-1^ in the OE-*OsNAS*/IR64 progeny, and 36 μg Fe g^-1^ and 56 μg Zn g^1^ in the OE-*OsNAS*/Esp progeny, were associated with significant reductions in a number of agro-morphological traits affecting grain yield. We identified grain-Zn and panicle-Mg as predictors accounting for the largest variation in agro-morphological traits in the OE-*OsNAS*/IR64 and OE-*OsNAS*/Esp progeny, respectively. The high-yield *japonica/japonica* OE-*OsNAS*/Esp progeny showed the largest increases in grain Fe and Zn with no overaccumulation of these metals in any other tissue, indicating that this genetic background may be better suited to tolerate the large Fe and Zn increases conferred by *OsNAS* overexpression. Our results indicate that it is readily possible to develop high-yielding rice lines with Fe and Zn biofortified polished grain using *OsNAS2* overexpression and by monitoring for grain Zn overaccumulation.

## Author Contributions

LM-M and AJ conceived the study and its design and drafted the manuscript. LM-M carried out the experimental activities and data analysis. JB conducted the transgene detection experiments. JB, JS-P, and JT participated in critical review of the manuscript.

## Conflict of Interest Statement

The authors declare that the research was conducted in the absence of any commercial or financial relationships that could be construed as a potential conflict of interest.

## Footnotes

1 http://www.unicef.org/
