# Supplementary material for: Association of Increased Grain Iron and Zinc Concentrations with Agro-morphological Traits of Biofortified Rice
Source: Front Plant Sci. 2016 Sep 28;7:1463. doi: 10.3389/fpls.2016.01463 (PMC5039209; doi:10.3389/fpls.2016.01463)
Supplement: Supplementary file 1 [file Table_1.DOCX]

| Plant tissue | Cross | Progeny type | Concentration (ug g^-1^ DW) | | | | | | | |
| --- | --- | --- | --- | --- | --- | --- | --- | --- | --- | --- |
|  |  |  | Fe | Zn | Mn | Cu | Ca | Mg | K | P |
| Root | OE-*OsNAS*/IR64 | NS | 6267 | 19 | 24 | 4 | 287 | 723 | 20533 | 4067 |
|  |  | + HY | 7350 | 19 | 24 | 7 | 308 | 748 | 22267 | 5133 |
|  |  | + LY | 9150 | 23 | 29 | 9 | 400 | 740 | 22600 | 6250 |
|  | OE-*OsNAS*/Es | NS | 8000 | 25 | 26 | 8 | 420 | 450 | 16250 | 4950 |
|  |  | + HY | 8120 | 43 | 28 | 9 | 496 | 539 | 18330 | 5350 |
|  |  | + LY | 6333 | 59 | 29 | 5 | 520 | 580 | 24167 | 5633 |
| Stem/ | OE-*OsNAS*/IR64 | NS | 273 | 8 | 124 | 4 | 1507 | 3800 | 27000 | 5767 |
| sheath |  | + HY | 258 | 12 | 152 | 4 | 1625 | 4317 | 31500 | 6467 |
|  |  | + LY | 283 | 26 | 228 | 7 | 1668 | 4200 | 30250 | 7150 |
|  | OE-*OsNAS*/Es | NS | 207 | 9 | 165 | 5 | 2250 | 4200 | 45000 | 5250 |
|  |  | + HY | 234 | 19 | 138 | 6 | 1431 | 3530 | 43300 | 6090 |
|  |  | + LY | 243 | 16 | 132 | 5 | 1303 | 3733 | 36000 | 7067 |
| Non- | OE-*OsNAS*/IR64 | NS | 237 | 10 | 477 | 8 | 15233 | 7167 | 16933 | 5133 |
| flag |  | + HY | 251 | 11 | 402 | 9 | 11400 | 7200 | 18317 | 4783 |
| leaf |  | + LY | 253 | 16 | 470 | 13 | 9125 | 5650 | 20400 | 5275 |
|  | OE-*OsNAS*/Es | NS | 295 | 13 | 470 | 9 | 11000 | 6750 | 19350 | 3250 |
|  |  | + HY | 337 | 14 | 439 | 12 | 8430 | 8000 | 21160 | 3730 |
|  |  | + LY | 423 | 10 | 383 | 12 | 9067 | 9500 | 20100 | 4233 |
| Flag | OE-*OsNAS*/IR64 | NS | 205 | 10 | 527 | 9 | 20000 | 4933 | 12167 | 4333 |
| leaf |  | + HY | 213 | 11 | 510 | 11 | 16650 | 4950 | 13333 | 3933 |
|  |  | + LY | 193 | 17 | 585 | 16 | 12050 | 4175 | 16900 | 4450 |
|  | OE-*OsNAS*/Es | NS | 295 | 15 | 705 | 11 | 15400 | 5350 | 13800 | 2500 |
|  |  | + HY | 296 | 18 | 694 | 16 | 11910 | 6630 | 15350 | 3230 |
|  |  | + LY | 320 | 14 | 420 | 15 | 10433 | 7533 | 14333 | 4000 |
| Panicle | OE-*OsNAS*/IR64 | NS | 89 | 17 | 165 | 8 | 3633 | 3367 | 9900 | 2667 |
|  |  | + HY | 107 | 11 | 150 | 8 | 2602 | 2947 | 10383 | 1933 |
|  |  | + LY | 122 | 35 | 140 | 13 | 1598 | 2800 | 9900 | 2060 |
|  | OE-*OsNAS*/Es | NS | 155 | 10 | 124 | 8 | 3800 | 2185 | 20350 | 1755 |
|  |  | + HY | 157 | 15 | 126 | 11 | 2586 | 2491 | 16110 | 2635 |
|  |  | + LY | 173 | 16 | 112 | 12 | 2457 | 4767 | 10433 | 3800 |
| Grain | OE-*OsNAS*/IR64 | NS | 14 | 15 | 17 | 4 | 165 | 1660 | 3267 | 4400 |
|  |  | + HY | 18 | 23 | 19 | 5 | 154 | 1560 | 3167 | 4217 |
|  |  | + LY | 29 | 56 | 23 | 9 | 137 | 1711 | 3444 | 4629 |
|  | OE-*OsNAS*/Es | NS | 16 | 20 | 30 | 6 | 179 | 1860 | 4550 | 5450 |
|  |  | + HY | 29 | 38 | 32 | 6 | 141 | 1882 | 4500 | 5470 |
|  |  | + LY | 36 | 63 | 28 | 6 | 127 | 2033 | 4067 | 5700 |

**Supplementary Table 1.** Concentration of 8 mineral nutrients in six plant tissues of the OE-*OsNAS*/IR64 and OE-*OsNAS*/Es progenies. NS, null segregants; +HY, transgenic high-yield; +LY, transgenic low-yield.
