# Supplementary material for: Association of Increased Grain Iron and Zinc Concentrations with Agro-morphological Traits of Biofortified Rice
Source: Front Plant Sci. 2016 Sep 28;7:1463. doi: 10.3389/fpls.2016.01463 (PMC5039209; doi:10.3389/fpls.2016.01463)
Supplement: Supplementary file 2 [file Table_2.DOCX]

**Supplementary Table 2.** Plant tissue nutrient predictors and agro-morphological traits used in the distance-based linear model (DistLM). A total of 48 predictors (8 nutrients assessed in 6 plant organs) were used in the DistLM analyses. Nine and four agro-morphological traits in the OE-*OsNAS*/IR64 and OE-*OsNAS*/Esp progeny were used in the DistLM analyses, respectively. DW, dry weight.

| Predictors | |  | Agro-morphological traits | |
| --- | --- | --- | --- | --- |
| Plant tissue | Nutrient |  | OE-*OsNAS*/IR64 | OE-*OsNAS*/Esp |
| Root | Fe |  | - Culm number | - Days to 50% flowering |
| Stem/Sheath | Zn |  | - Plant height | - Spikelet fertility |
| Non-flag leaf | Cu |  | - Number of filled grain per  main panicle | - Number of filled grain per main  panicle |
| Flag-leaf | Mn |  | - Total grain per main panicle | - Estimated grain yield per plant |
| Panicle | K |  | - Spikelet fertility |  |
| Grain | Mg |  | - Estimated grain yield per plant |  |
|  | Ca |  | - Root DW |  |
|  | P |  | - Stem/sheath DW |  |
|  |  |  | - Panicle DW |  |
