# Supplementary material for: Association of Increased Grain Iron and Zinc Concentrations with Agro-morphological Traits of Biofortified Rice
Source: Front Plant Sci. 2016 Sep 28;7:1463. doi: 10.3389/fpls.2016.01463 (PMC5039209; doi:10.3389/fpls.2016.01463)
Supplement: Supplementary file 3 [file Table_3.DOCX]

**Supplementary Table 3.** Agro-morphological performance of transgenic progeny classified according to their *OsNAS* overexpression construct. Values given are means. No statistical test was performed in the *OE-OsNAS/IR64* progeny due to the small sample size for the OE-*OsNAS2* group.

| Construct | Days to 50% flowering | Culm No | Plant height (cm) | Panicle length (cm) | No of filled grain per main panicle | Total grain per main panicle | Spikelet fertility (%) | Estimated grain yield per plant (g) | Dry weight (g) | | | | |
| --- | --- | --- | --- | --- | --- | --- | --- | --- | --- | --- | --- | --- | --- |
|  |  |  |  |  |  |  |  |  | Root | Stem/ sheath | Non-flag leaf | Flag  leaf | Panicle |
| *OE-OsNAS/IR64* |  |  |  |  |  |  |  |  |  |  |  |  |  |
| OE-*OsNAS1* (n=8) | 102 | 6 | 72.1 | 22.5 | 57 | 105 | 51.26 | 5.84 | 1.74 | 6.42 | 2.90 | 0.71 | 0.29 |
| OE-*OsNAS2* (n=2) | 97 | 9 | 102.3 | 26.5 | 89 | 113 | 76.65 | 13.43 | 4.08 | 20.55 | 5.17 | 1.51 | 0.60 |
| NS (n=3) | 99 | 11 | 92.2 | 25.4 | 110 | 153 | 72.22 | 20.90 | 3.80 | 17.07 | 5.13 | 1.18 | 0.65 |
| *OE-OsNAS/Esp* |  |  |  |  |  |  |  |  |  |  |  |  |  |
| OE-*OsNAS1* (n=9) | 98 | 4 | 90.6 | 19.7 | 75 | 105 | 69.96 | 6.19 | 1.96 | 6.26 | 2.55 | 0.50 | 0.21 |
| OE-*OsNAS2* (n=4) | 100 | 4 | 100.5 | 23.4 | 69 | 102 | 70.43 | 5.38 | 1.57 | 5.69 | 2.08 | 0.37 | 0.20 |
| NS (n=3) | 94 | 4 | 102.8 | 22.8 | 126 | 140 | 88.39 | 9.97 | 1.52 | 6.39 | 2.08 | 0.43 | 0.26 |
| *P*-value _(construct)_ | n.s. | n.s. | n.s. | n.s. | n.s. | n.s. | n.s. | n.s. | n.s. | n.s. | n.s. | n.s. | n.s. |

n.s., Not significant.
