# Supplementary material for: Association of Increased Grain Iron and Zinc Concentrations with Agro-morphological Traits of Biofortified Rice
Source: Front Plant Sci. 2016 Sep 28;7:1463. doi: 10.3389/fpls.2016.01463 (PMC5039209; doi:10.3389/fpls.2016.01463)
Supplement: Supplementary file 4 [file Image_1.PDF]

A B

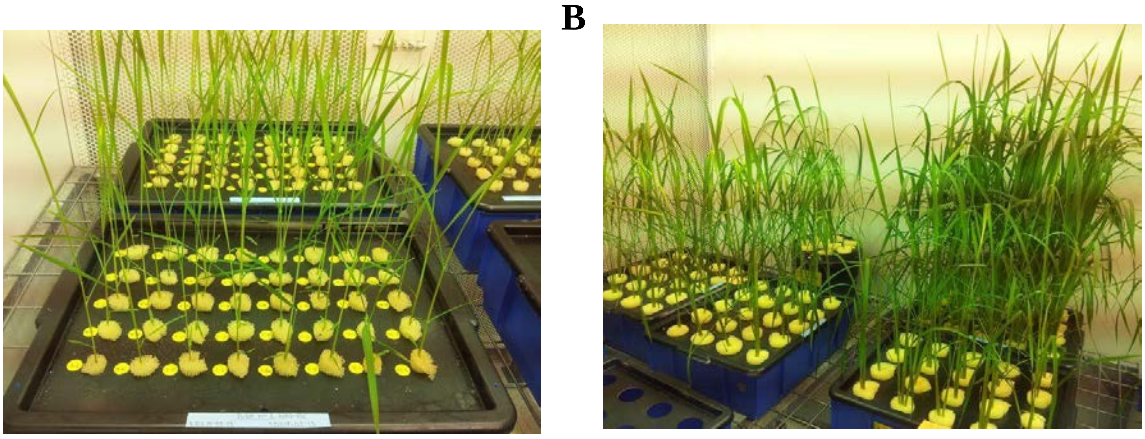

Supplementary Figure 1. Hydroponic set up for cultivation of plants from the OE-*OsNAS*/IR64 and OE-*OsNAS*/Esp progenies. (**A**) Culture boxes covered with removable lids of 40 holes of 25mm in diameter. (**B**) Culture boxes covered with removable lids of 20 holes of 50 mm in diameter.
