# Supplementary material for: Association of Increased Grain Iron and Zinc Concentrations with Agro-morphological Traits of Biofortified Rice
Source: Front Plant Sci. 2016 Sep 28;7:1463. doi: 10.3389/fpls.2016.01463 (PMC5039209; doi:10.3389/fpls.2016.01463)
Supplement: Supplementary file 5 [file Image_2.PDF]

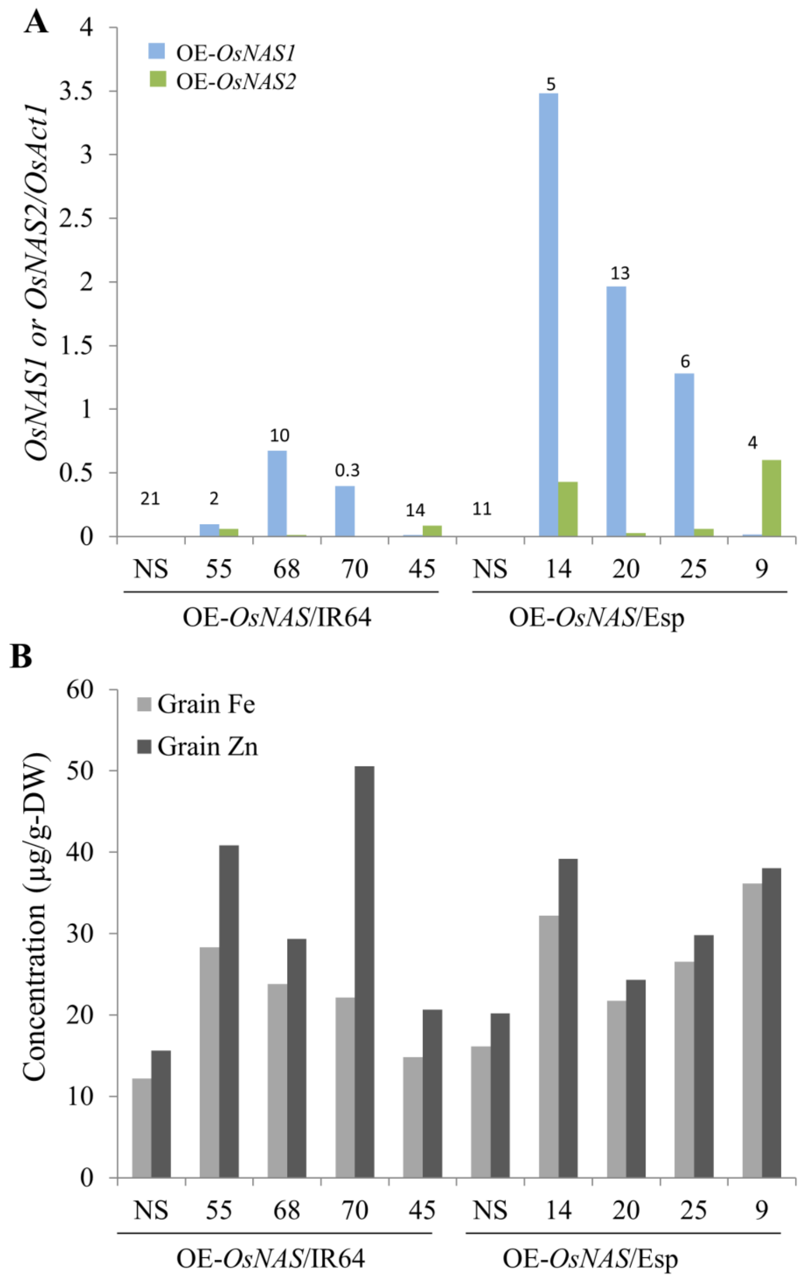

Supplementary Figure 2. Relationship between (**A**) *OsNAS1/2* gene expression and (**B**) grain Fe and Zn concentrations of OE-*OsNAS*/IR64 and OE-*OsNAS*/Esp progenies. The *OsNAS1* or *OsNAS2* gene expression was obtained by semi-quantitative RT-PCR relative to housekeeping *OsAct1* gene expression on a 1% agarose gel stained with ethidium bromide. Values represent estimated grain yield per plant (g).
