# Supplementary material for: Association of Increased Grain Iron and Zinc Concentrations with Agro-morphological Traits of Biofortified Rice
Source: Front Plant Sci. 2016 Sep 28;7:1463. doi: 10.3389/fpls.2016.01463 (PMC5039209; doi:10.3389/fpls.2016.01463)
Supplement: Supplementary file 6 [file Image_3.PDF]

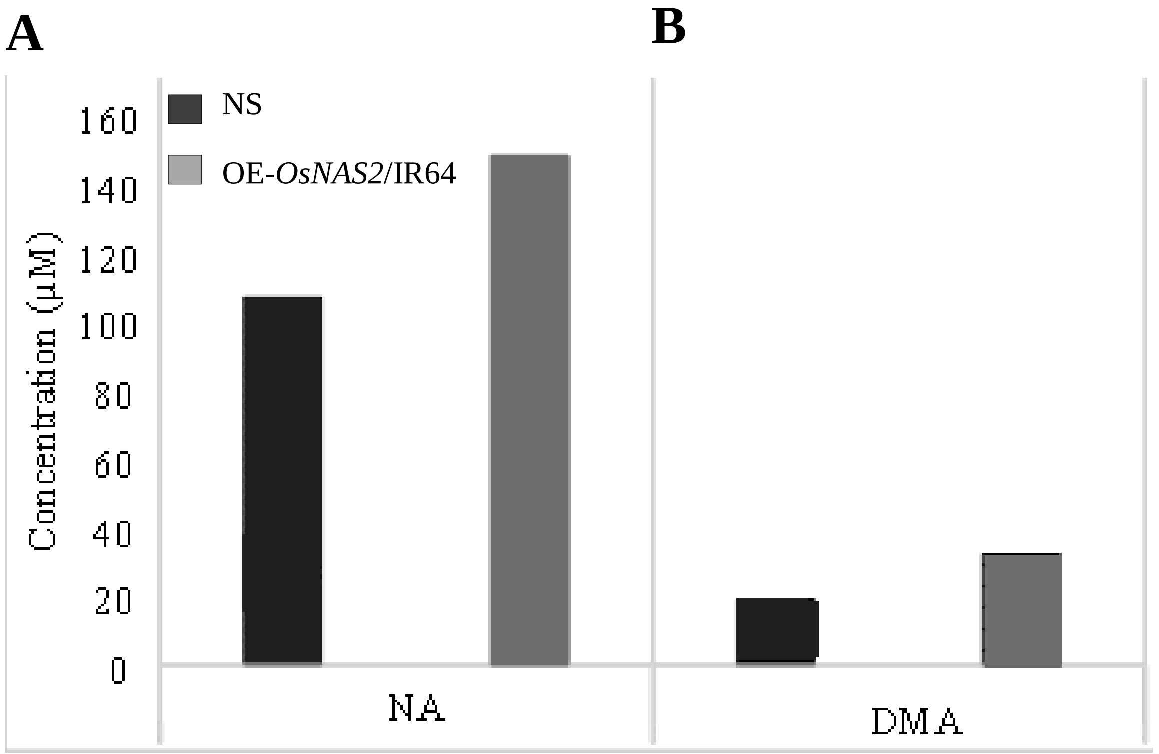

Supplementary Figure 3. Concentrations of (**A**) NA and (**B**) DMA in the xylem sap bulks of 10 mature null segregants (NS) and 10 OE-*OsNAS*2/IR64 progenies. NA and DMA concentrations were quantified by Liquid chromatography triple quadrupole mass spectrometry (LC-qqq-MS). Values presented are the mean concentration (µM) of 2 technical replicates. Data provided by Jamie Selby-Pham.
