# Supplementary material for: Association of Increased Grain Iron and Zinc Concentrations with Agro-morphological Traits of Biofortified Rice
Source: Front Plant Sci. 2016 Sep 28;7:1463. doi: 10.3389/fpls.2016.01463 (PMC5039209; doi:10.3389/fpls.2016.01463)
Supplement: Supplementary file 7 [file Image_4.PDF]

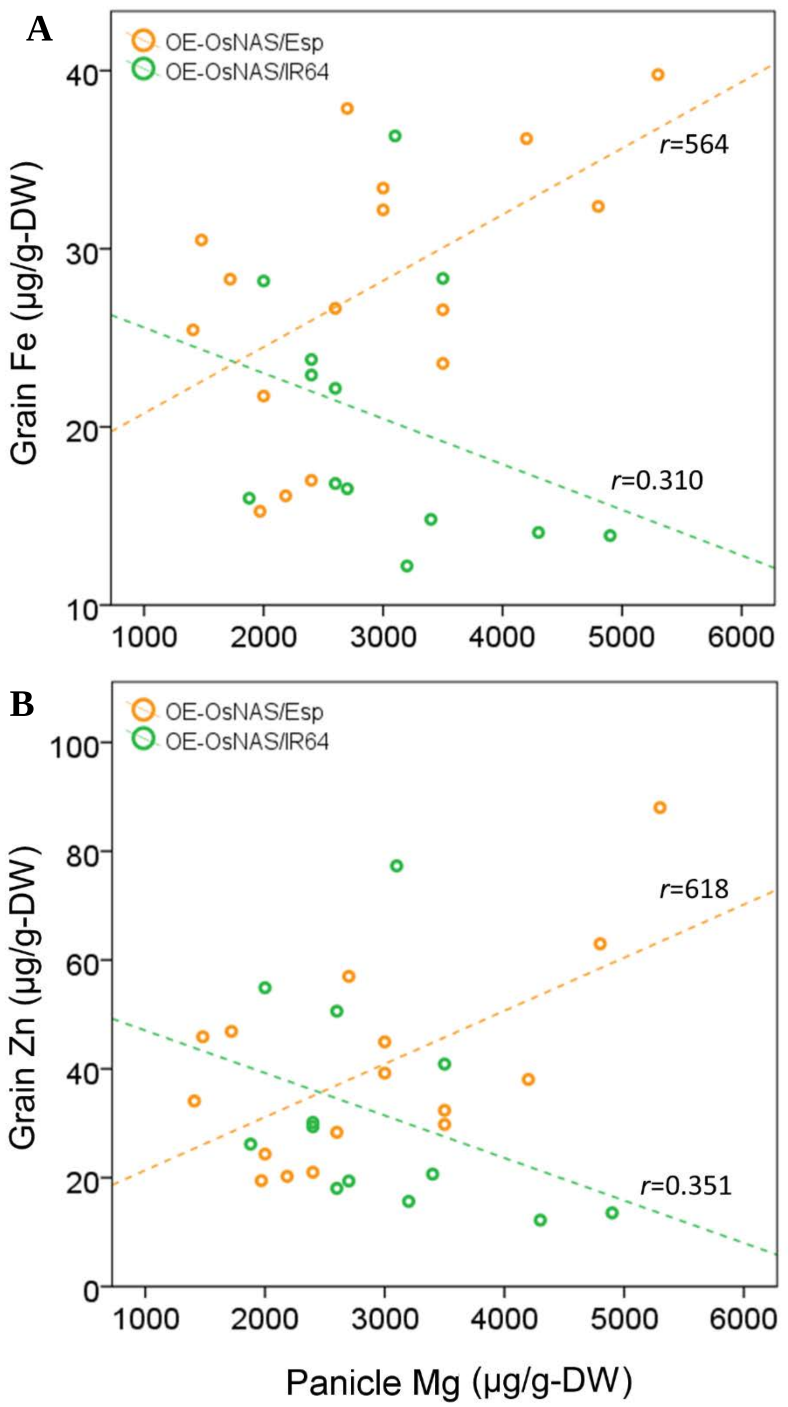

Supplementary Figure 4. Linear correlation between **(A)** grain Fe and panicle Mg, and **(B)** grain Zn and panicle Mg for the OE-*OsNAS*/IR64 and OE-*OsNAS*/Esp progenies.
